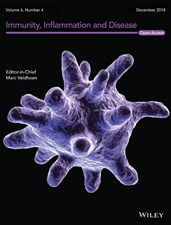# Issue Information

**DOI:** 10.1002/iid3.196

**Published:** 2018-11-21

## Abstract